# Double-frequency Aharonov-Bohm effect and non-Abelian braiding properties of Jackiw-Rebbi zero-mode

**DOI:** 10.1093/nsr/nwz189

**Published:** 2019-11-22

**Authors:** Yijia Wu, Haiwen Liu, Jie Liu, Hua Jiang, X C Xie

**Affiliations:** 1 International Center for Quantum Materials, School of Physics, Peking University, Beijing 100871, China; 2 Center for Advanced Quantum Studies, Department of Physics, Beijing Normal University, Beijing 100875, China; 3 Department of Applied Physics, School of Science, Xi’an Jiaotong University, Xi’an 710049, China; 4 School of Physical Science and Technology, Soochow University, Suzhou 215006, China; 5 Beijing Academy of Quantum Information Sciences, Beijing 100193, China; 6 CAS Center for Excellence in Topological Quantum Computation, University of Chinese Academy of Sciences, Beijing 100190, China

**Keywords:** Jackiw-Rebbi zero-mode, Aharanov-Bohm effect, non-Abelian statistics, Majorana zero-mode

## Abstract

Ever since its first proposal in 1976, Jackiw-Rebbi zero-mode has been drawing extensive attention for its charming properties including charge fractionalization, topologically protected zero-energy and possible non-Abelian statistics. We investigate these properties through the Jackiw-Rebbi zero-modes in quantum spin Hall insulators. Though charge fractionalization is not manifested, Jackiw-Rebbi zero-mode's zero-energy nature leads to a double-frequency Aharonov-Bohm effect, implying that it can be viewed as a special case of Majorana zero-mode without particle-hole symmetry. Such relation is strengthened for Jackiw-Rebbi zero-modes also exhibiting non-Abelian properties in the absence of superconductivity. Furthermore, in the condition that the degeneracy of Jackiw-Rebbi zero-modes is lifted, we demonstrate a novel non-Abelian braiding with continuously tunable fusion rule, which is a generalization of Majorana zero-modes’ braiding properties.

## INTRODUCTION

Jackiw-Rebbi zero-mode was first raised as the zero-energy soliton solution of Dirac equation in one spatial dimension [1]. In the presence of such a zero-mode, the total charge of the ‘Dirac sea’ is half-integer [2–6] due to charge-conjugation symmetry, which is regarded as another mechanism of charge fractionalization, in addition to the prestigious fractional quantum Hall (FQH) effect [7–10]. In condensed matter physics, Jackiw-Rebbi zero-mode is closely related to the band topology [11,12]. The first famous example is the Su-Schrieffer-Heeger (SSH) model [13] whose low-energy effective Hamiltonian is equivalent to a 1D topological insulator (TI), and Jackiw-Rebbi zero-mode resides in the domain wall separating topologically distinct phases. Another celebrated example is the Kitaev's chain [14] whose effective Hamiltonian is again equivalent to a 1D TI. The difference is that the zero-mode here is self-conjugate due to the superconductivity and therefore a Majorana one. In this vein, Jackiw-Rebbi zero-mode can be regarded as a special case of Majorana zero-mode (MZM) in the absence of particle-hole (PH) symmetry [10,15].

In the last decade, Jackiw-Rebbi zero-mode was proposed in topological systems including spin ladders [16,17], Rashba nanowires [18–21], and quantum spin Hall insulator (QSHI) with constriction [22,23] or external magnetic field [24]. These zero-modes are created or annihilated pairwisely, hence the braiding processes are non-commutative [8] due to the fermion parity conservation [16,25]. However, on the contrary of its Majorana cousin [26], Jackiw-Rebbi zero-mode's non-Abelian nature has not yet been demonstrated in a practical device such as trijunction [17,27–30] or cross-shaped junction [31,32] as has been done for MZMs.

Another peculiar property of the Jackiw-Rebbi zero-mode is the 1/2 charge fractionalization, which has been claimed [24] to be detectable in a pumping process [33] or by Coulomb blockade. Recently, a novel 3/2 FQH plateau is observed in single layer 2D electron gas with confined geometry [34]. Jackiw-Rebbi zero-mode induced by the confined geometry could be a tentative explanation [34] that similar mechanism has been proposed in QSHI [22].

In this article, we first construct a QSHI heterostructure supporting Jackiw-Rebbi zero-mode. Then we investigate the Aharonov-Bohm (AB) effect where a single Jackiw-Rebbi zero-mode is embedded in an AB ring, showing a double-frequency AB oscillation at zero-energy. A comparison with MZM’s AB effect supports the aforementioned relation between Jackiw-Rebbi zero-mode and MZM. We also set up a cross-shaped QSHI junction and numerically confirm the non-Abelian braiding properties of Jackiw-Rebbi zero-modes to be in analogy with MZMs. Importantly, Jackiw-Rebbi zero-modes represent a novel non-Abelian braiding corresponding to a generalized fusion rule when the zero-modes’ degeneracy is lifted by chiral-symmetry-breaking disorder or by tuning gate voltages. Such generalized and continuously tunable non-Abelian braiding can also be realized for MZMs if ‘fictitious’ disorder breaking PH-symmetry is presented.

## LATTICE REALIZATION OF JACKIW-REBBI ZERO-MODE

The 1D effective Hamiltonian describing four edge channels of QSHI constriction can be constructed [22] as ${H_{\rm 1D}} = {v_F}\ {\hat{p}_x}{\rho _z}{\tau _0} + {{\rm{\Delta }}_x}{\rho _x}{\tau _0} + {{\rm{\Delta }}_z}{\rho _z}{\tau _z} + t{\rho _x}{\tau _x}$, where the four terms represent the kinetic energy, the spin-orbit interaction (SOI), the Zeeman term, and a spin-conserved inter-edge tunneling term, respectively (${\rho _i}$, ${\tau _i}$ are Pauli matrices working in right-/left-moving spinor and chirality spinor, respectively). The competition between the Zeeman term ${{\rm{\Delta }}_z}$ and the tunneling strength *t* induces two distinct topological phases and the Jackiw-Rebbi zero-mode resides in the domain wall. The effective Hamiltonian ${H_{\rm{eff}}} = \ ( {{{\rm{\Delta }}_x}/t} ){p_x}{\pi _x} + ( {{{\rm{\Delta }}_z} - t} ){\pi _z}$ describing topological phase transition has the form of 1D TI (${\pi _i}$ are Pauli matrices for real spin). It is worth noting that quantum Hall insulator with two pairs of edge channels [34] possesses similar Hamiltonian.

**Figure 1.**
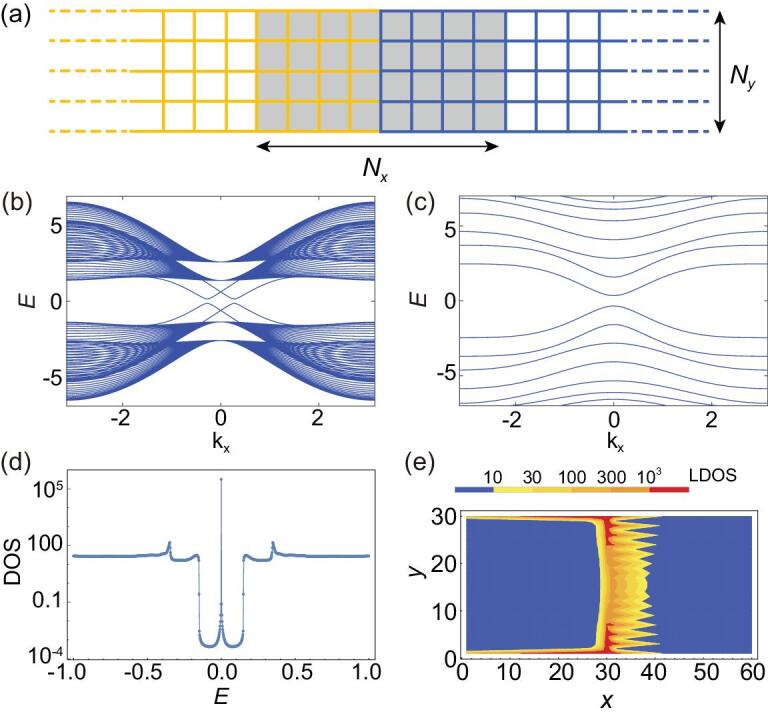
(a) Schematic diagram of the QSHI heterostructure lattice [with finite width ${N_y} = \ 30$ through (a-e)] which is composed of two semi-infinite QSHI halves (yellow and blue, respectively) whose energy spectrums are shown as (b) and (c), respectively. Hamiltonian parameter ${A_y} = \ 2$ in (b) and there are four edge channels; ${A_y} = \ 40$ in (c) and the edge channels here are destroyed. (d) The DOS inside the shaded region (${N_x} = \ 60$) of (a). (e) The local density of states distribution inside the shaded region of (a) at energy $E\ = \ 0$. Other parameters are ${A_x} = \ 2.2$, $B\ = \ 1$, $M\ = \ 2$, ${{\rm{\Delta }}_z} = \ 0.6$, and ${{\rm{\Delta }}_x} = \ 0.15$.

The 2D Hamiltonian of QSHI constriction supporting Jackiw-Rebbi zero-modes is obtained by adding the Zeeman term ${{\rm{\Delta }}_z}$ and the SOI term ${{\rm{\Delta }}_x}$ into the Bernevig-Hughes-Zhang (BHZ) model [35,36] as:

(1) \begin{equation*}{H_{\rm QSHI}}\left( {\bf p} \right){\rm{\ }} {=} \left( {\begin{array}{@{}*{2}{c}@{}} {h\! \left( {\bf p} \right) + {{\rm{\Delta }}_z}{\sigma _0}}&{{{\rm{\Delta }}_x}{\sigma _0}}\\ {{{\rm{\Delta }}_x}{\sigma _0}}&{{h^{\rm{*}}}\left( { {-} {\bf p}} \right) {-} {{\rm{\Delta }}_z}{\sigma _0}} \end{array}} \right){\rm{\ }}, \end{equation*}

where $h( {\bf p} )\ = \ ( {{A_x}{p_x}{\sigma _x} - {A_y}{p_y}{\sigma _y}} ) + ( {M - B{{\bf p}^2}} ){\sigma _z}$ (${\sigma _i}$ for Pauli matrices). The discretized version of Equation (1) in a 2D lattice with finite width ${N_y}$ generally possesses four edge states. However, a strong inter-edge tunneling will destroy these edge states and bring about a topologically trivial phase. The strength of such tunneling depends on the overlap between opposite edge states, therefore it can be modulated through the Hamiltonian parameters [e.g., ${A_y}$, see Fig. 1(b, c)] or lattice width ${N_y}$ (i.e., QSHI constrictions [22,23]). Based on the method of Green's function, the density of states (DOS) [Fig. 1(d, e)] of a QSHI heterostructure [Fig. 1(a)] composed of two topologically distinct halves demonstrates the subgap zero-energy Jackiw-Rebbi state localized at its interface. It is worth noting that the Jackiw-Rebbi zero-mode's energy may deviate from zero [e.g. if kinetic term ($C - D{{\bf p}^2}$) is presented in the BHZ model]. However, both the transport and the braiding properties of the Jackiw-Rebbi zero-modes remain unchanged in the presence of such a constant shift of energy.

## THE AHARONOV-BOHM EFFECT OF JACKIW-REBBI ZERO-MODE AND A COMPARISON WITH MZM

**Figure 2.**
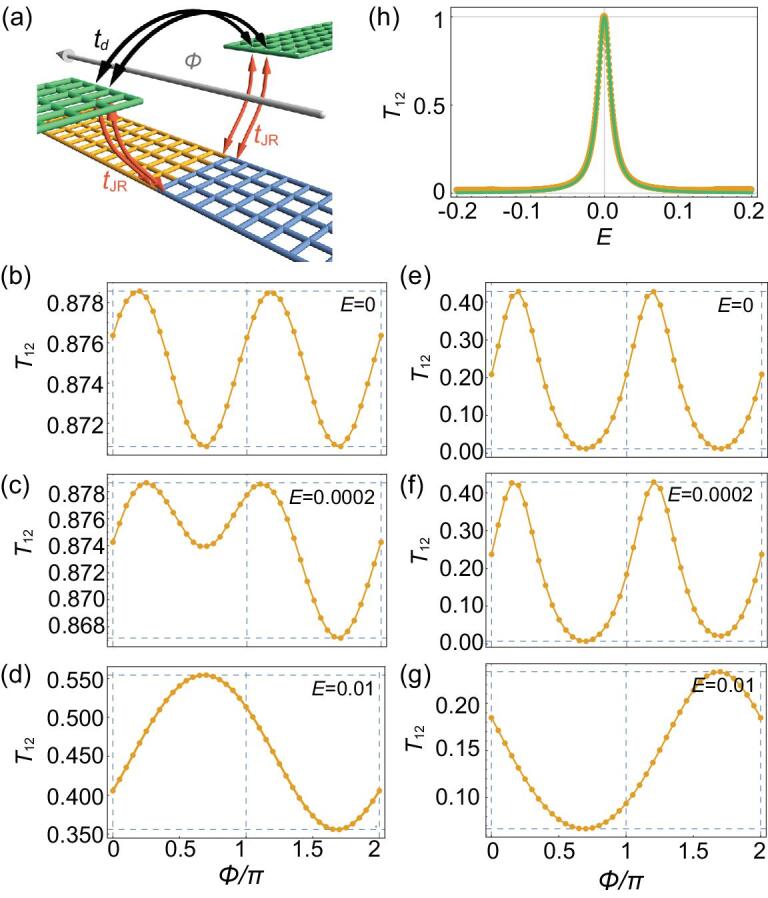
(a) Sketch of the AB ring in a lattice model. An AB ring enclosing a magnetic flux ${\phi}$ is sandwiched between two identical 2D metal leads (green lattice). The upper arm of the ring is the direct hopping with strength ${{{t}}_{{d}}}$ between the two leads (black arrows), and the lower arm contains a Jackiw-Rebbi zero-mode in a QSHI heterostructure (yellow and blue lattice) which is equally coupled to the two leads with hopping strength ${{{t}}_{{\rm{JR}}}}$ (orange arrows). (b-d) [(e-g)] In the condition of weak (strong) ${{{t}}_{{d}}}$, numerical results of ${{{T}}_{12}}$ at fixed energies as (b) [(e)] ${E} \,=\, 0$, (c) [(f)] ${E} \,=\, 0.0002$, and (d) [(g)] ${E} \,=\, 0.01$, respectively. For simplicity, in (b-g), conductance constants irrelevant to the oscillations have been subtracted. (h) ${{T}_{12}}$ solely induced by the Jackiw-Rebbi zero-mode, where numerical results (orange) can be perfectly fitted by the analytic formula (green) with ${{\tilde{E}}} \approx 95{{E}}$.

Apart from pumping of the domain wall [24,33], transport signature of Jackiw-Rebbi zero-mode is also shown in the Jackiw-Rebbi zero-mode intermediated electron transmission [18,37], whose peculiarities may be revealed by a two-path interference with a normal electron transmission, i.e., AB effect with a Jackiw-Rebbi zero-mode embedded [Fig. 2(a)]. The direct hopping strength between the two leads is ${t_d}$, and the hopping strength between the leads and the QSHI heterostructure supporting Jackiw-Rebbi zero-mode is ${t_{\rm JR}}$. The transmission coefficient between the two leads ${T_{12}}$ depending on the incident electron's energy *E* as well as the magnetic flux $\phi $ inclosed [$\phi $ is in the unit of ${\phi _0}/( {2\pi } )$, ${\phi _0}$ is the flux quantum] can be numerically investigated by the Green's function. For weak ${t_d}$ [Fig. 2(b-d)], the AB effect shows an unexpected $\pi $-period sinusoidal oscillation in the zero-energy case that the incident electron's energy *E* matches the zero-mode's energy level. As *E* slightly deviates from zero, ${T_{12}}$ becomes the superposition of a $\pi $-period and a $2\pi $-period sinusoidal functions. The AB effect comes back to the normal $2\pi $-period sinusoidal oscillation for significant non-zero *E*. For strong ${t_d}$ [Fig. 2(e-g)], though no longer in the simple sinusoidal form, the AB effect still exhibits a $\pi $-period oscillation for $E\ = \ 0$, while a $2\pi $-period oscillation for $E \ne 0$.

The *S*-matrix theory [38,39] shows that ${T_{12}}$ has the analytical form as:

(2) \begin{equation*}{T_{12}} = \frac{{4\tilde{t}_d^2 \cdot {{\tilde{E}}^2} + 4{{\tilde{t}}_d} \,{\rm cos} {\rm{\ }}\phi \cdot \tilde{E} + 1}}{{{{\left[ {\left( {1 + \tilde{t}_d^2} \right)\tilde{E} + {{\tilde{t}}_d}\,{\rm cos} {\rm{\ }}\phi } \right]}^2} + 1}}{\rm{\ }}, \end{equation*}

where ${\tilde{t}_d} \equiv \frac{{{t_d}}}{{2{v_f}}}\ $, and $\tilde{E} \equiv \frac{{{v_f}}}{{t_0^2}}( {E - {\epsilon _0}} )$ (see Supplementary Material). In the resonant tunneling condition $\tilde{E} = \ 0$, ${T_{12}}$ reduces to $1/( {\tilde{t}_d^2\mathop {\cos }^2 \phi + 1)} $ thus verifies the $\pi $-period AB oscillation. Furthermore, the numerical results can be well fitted by Equation (2) (see Supplementary Material). In the limit of ${\tilde{t}_d} \to 0$, ${T_{12}} = \ 1/( {{{\tilde{E}}^2} + 1} )$ indicates the transmission solely induced by the Jackiw-Rebbi zero-mode has a peak value of 1, other than the naively expected $1/2$. Such analytic result drawn from *S*-matrix is numerically verified [Fig. 2(h)] and consistent with previous research [18].

For comparison, if the Jackiw-Rebbi zero-mode in the AB ring is replaced by a MZM [40,41], then the transmission conductance ${G_{12}}$ between the two leads is related to the *S*-matrix in the Bogoliubov-de Gennes basis [38,42] as

(3) \begin{equation*}\begin{array}{@{}*{2}{l}@{}} {{G_{12}}}&{ = \frac{{{e^2}}}{h}\ \cdot \left\{ {\left| {S_{12}^{ee}{|^2} - } \right|S_{12}^{he}{|^2}} \right\}}\\ {}&{ = \frac{{{e^2}}}{h} \cdot \frac{{ - 32{{\tilde{t}}_d}\sin \,\phi + 8{{\tilde{t}}_d}\left( {1 - \tilde{t}_d^2} \right)\cos \,\phi \cdot \tilde{E} + 4\tilde{t}_d^2\left( {1 + \tilde{t}_d^2} \right) \cdot {{\tilde{E}}^2}}}{{\left( {1 + \tilde{t}_d^2} \right) \cdot {{[ {16 + (1 + \tilde{t}_d^2} )}^2} \cdot {{\tilde{E}}^2}}] }}, \end{array}\end{equation*}

where $1,2$ for lead indices, $e,\ h$ for electron and hole, respectively, ${\tilde{t}_d} \equiv \frac{{{t_d}}}{{2{v_f}}}$, and $\tilde{E} \equiv \frac{{2{v_f}}}{{t_M^2}}E$ (see Supplementary Material). In contrast to the Jackiw-Rebbi zero-mode, Equation (3) indicates that MZM’s AB effect oscillates in a $2\pi $-period at both zero-bias ($\tilde{E} = \ 0$) and finite-bias ($\tilde{E} \ne 0$) [40]. However, as discussed above, Jackiw-Rebbi zero-mode can be viewed as a special case of MZM where PH symmetry is absent. The electron and hole indices in Equation (3) are replaced by two electron subband indices (denoted by $\alpha $ and $\beta $) if the Majorana condition is not imposed. Hence the sign difference between electron and hole is absent, and Equation (3) is modified as ${G_{12}} = {{{e^2}} \over h}\{ {| {S_{12}^{\alpha \alpha }{|^2} + } |S_{12}^{\beta \alpha }{|^2}} \} = {{{e^2}} \over h}\,\,{{16} \over {16 + {{( {1 + \tilde t_d^2} )}^2} \cdot {{\tilde E}^2}}}.\{ {{1 \over 2} \,+\, {{{\tilde t_d^2} \over {{\rm{}} (1 + \tilde t_d^2)}^2}}}\,\, +\, {{\tilde t_d^2} \over 4}{{\tilde E}^2}\, +\, {{{{\tilde t}_d}} \over 2}{{1 - \tilde t_d^2} \over {1\,\, +\,\, \tilde t_d^2}}\\ {\rm{cos\,\,}} \phi \cdot \tilde E - {{\tilde t_d^2} \over {{{( {1 + \tilde t_d^2} )}^2}}}{\rm{cos\,\,}}2\phi \}$, which qualitatively retrieve the consequence that the Jackiw-Rebbi zero-mode exhibits $\pi $-period ($2\pi $-period) AB effect at zero-bias (finite-bias).

## NON-ABELIAN BRAIDING PROPERTIES

**Figure 3.**
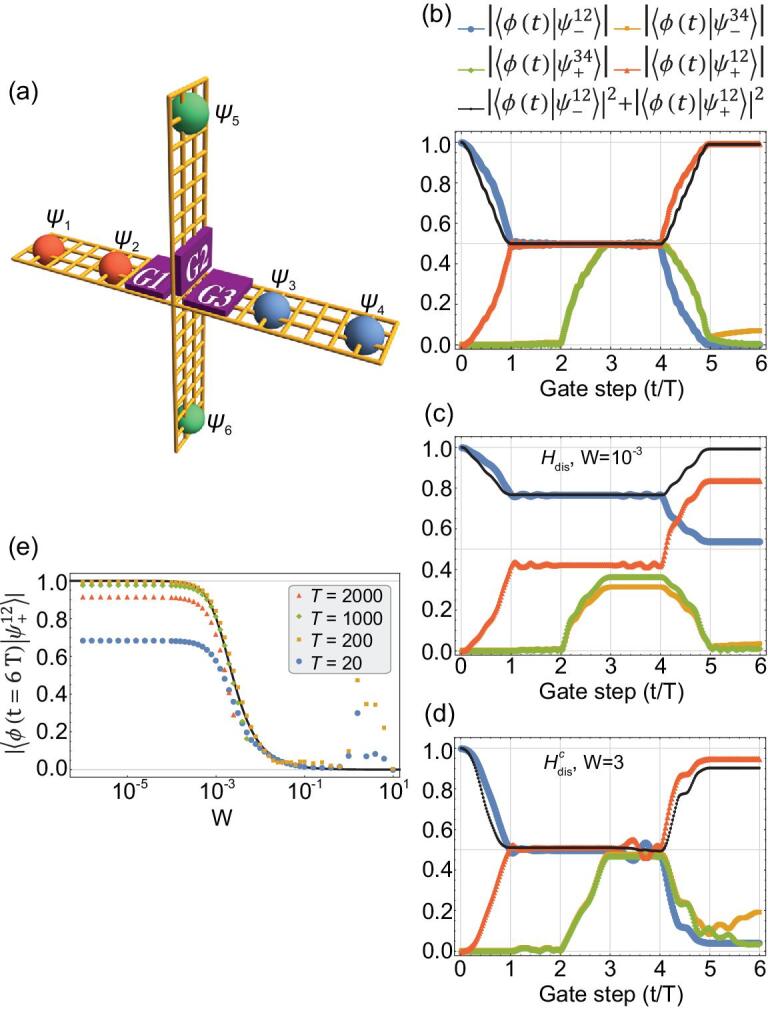
(a) Sketch of the cross-shaped QSHI junction. Distributions of the three pairs of Jackiw-Rebbi zero-modes (${{{\psi }}_{{{i\ }} = {{\ }}1,{{\ }}2,{{\ }} \ldots ,{{\ }}6}}$) before braiding are shown. (b-d) Evolution of an eigenstate ${{\phi }}( {{t}} )$ as ${{{\psi }}_2}$ and ${{{\psi }}_3}$ are swapped twice in succession with ${{T\ }} = {{\ }}200$ (b) in the clean limit; (c) in the presence of chiral-symmetry-breaking disorder ${{{H}}_{{\rm{dis}}}}$ with ${{W\ }} = {{\ }}{10^{ - 3}}$; (d) in the presence of chiral-symmetry-conserved disorder ${{H}}_{{\rm{dis}}}^{{C}}$ with ${{W\ }} = {{\ }}3$. ${| {\langle {{{\phi }}( {{{t\ }} = {{\ }}6{{T}}} )} |{{\psi }}_ - ^{12}\rangle} |^2} + {{\ }}{| {\langle {{{\phi }}( {{{t\ }} = {{\ }}6{{T}}} )} |{{\psi }}_ + ^{12} \rangle } |^2} = {{\ }}1$ is valid in (b) and (c), while invalid in (d) for strong disorder destructing topological gap. (e) $| {\langle {{{\phi }}( {{{t\ }} = {{\ }}6{{T}}} )} |{{\psi }}_ + ^{12} \rangle } |$ in a fixed chiral-symmetry-breaking disorder profile with different disorder strength *W* and braiding time *T*. Numerical results can be well fitted (black curve) by Equation (5) as $| {\langle {{{\phi }}( {{{t\ }} = {{\ }}6{{T}}} )} |{{\psi }}_ + ^{12} \rangle } | = {{\ }}1/{({\tilde{\bf \Delta }}_{12}^2 + 1)^{1/2}}$ for intermediate *T* satisfying adiabatic condition ${{{\bf \Delta }}_{{b}}} \gg 1/{{T}} \gg {{{\epsilon }}_{12}},{{\ }}{{{\epsilon }}_{34}}$. Topological gap ${{{\bf \Delta }}_{{b}}} \approx 0.2$, the coupling energy ${{{\epsilon }}_{12}},{{\ }}{{{\epsilon }}_{34}} \approx 7 \times {10^{ - 5}}$.

The similarities between Jackiw-Rebbi and MZM revealed by the AB effect inspire us to investigate the possible non-Abelian statistics of Jackiw-Rebbi zero-modes through cross-shaped junction [Fig. 3(a)]. Each of the four arms of the junction is a topologically nontrivial QSHI supporting Jackiw-Rebbi zero-modes, and three gates (G1, G2, and G3) are located near the crossing. If the gate voltage is turned on (off), then the corresponding arm is separated (connected) due to the presence (absence) of the gating potential barrier. Initially, G1 and G3 are turned on while G2 is turned off, hence three pairs of Jackiw-Rebbi zero-modes (${\psi _1},\ {\psi _2},\ \ldots {\psi _6}$) are localized at the ends of the three divided parts [Fig. 3(a)]. The braiding protocol [31] takes three steps (time cost for each step is *T*) to swap ${\psi _2}$ and ${\psi _3}$ spatially. Firstly, G1 is turned off and then G2 is turned on, hence ${\psi _2}$ is moved to the top of G2. Secondly, G3 is turned off and then G1 is turned on, so now ${\psi _3}$ is at the left of G1. Thirdly, turning off G2 is followed by turning on G3, as a result ${\psi _2}$ and ${\psi _3}$ are swapped. In the whole braiding process taking time of $2\ \times \ 3T$, ${\psi _2}$ and ${\psi _3}$ are swapped twice in succession.

In the following, we verify that swapping ${\psi _2}$ and ${\psi _3}$ once lead to ${\psi _2} \to {\psi _3}$ and ${\psi _3} \to - {\psi _2}$, which is identical to the MZM [26,43]. In the clean limit, finite-size induced coupling betweem paired Jackiw-Rebbi zero-modes ${\epsilon _{2i - 1,2i}}{e^{i{\alpha _{2i - 1,2i}}}}\psi _{2i - 1}^\dagger {\psi _{2i}} + h.c$. ($i\ = \ 1,2,3$, and ${\alpha _{2i - 1,2i}}$ for arbitrary phase) leads to symmetric and antisymmetric eigenstates $\psi _ \pm ^{12} = \frac{1}{{\sqrt 2 }}\ ( {{\psi _1} \pm {e^{ - i{\alpha _{12}}}}{\psi _2}} )$ [16]. Numerical simulation of the adiabatical time-evolution (see Supplementary Material) shows that an eigenstate $\phi ( t )$ evolving from $\psi _ - ^{12}$ to $\psi _ + ^{12}$ after ${\psi _2}$ and ${\psi _3}$ are swapped twice in succession [Fig. 3(b)], which indicates ${\psi _2} \to - {\psi _2}$. Similarly, ${\psi _3} \to - {\psi _3}$ is confirmed for another eigenstate evolving from $\psi _ - ^{34}$ to $\psi _ + ^{34}$ simultaneously. Consequently, braiding properties ${\psi _2} \to {\psi _3}$ and ${\psi _3} \to - {\psi _2}$ (after swapping ${\psi _2}$ and ${\psi _3}$ once) can be drawn from the above results up to a gauge transformation. It is worth noting that the basis of the wavefunction we adopted here is $( {| {\psi _ - ^{12}} \rangle ,\ | {\psi _ - ^{34}} \rangle ,\ | {\psi _ + ^{34}} \rangle ,\ | {\psi _ + ^{12}} \rangle } )$ [Fig. 3(b)], while the basis usually chosen for MZMs’ braiding is $( {| 0 \rangle ,\ {\rm{\Psi }}_1^\dagger | 0 \rangle ,\ {\rm{\Psi }}_2^\dagger | 0 \rangle ,\ {\rm{\Psi }}_1^\dagger {\rm{\Psi }}_2^\dagger | 0 \rangle } )$ [26,31,32] instead.

## GENERALIZED NON-ABELIAN BRAIDING IN SYSTEMS WITH DEGENERACY LIFTING

The QSHI constriction [Equation (1)] is in the AIII symmetry class possessing chiral symmetry $- {H_{\rm QSHI}}( { - {\bf{p}}} )\ = \ {CH_{\rm QSHI}}( {\bf{p}} ){C^{ - 1}}$ [44,45], which protects the degeneracy of Jackiw-Rebbi zero-modes ($C\ = {\pi _y}\ {\sigma _y}$, ${\pi _y}$ is Pauli matrix for real spin). Strikingly, the eigenstate $\phi ( t )$ evolves from $\psi _{12}^ - $ to a superposition of $\psi _{12}^ - $ and $\psi _{12}^ + $ [Fig. 3(c)] in the presence of tiny chiral-symmetry-breaking disorder ${H_{\rm dis}}\,{=}\,{\rm diag}\{ {{V_1}( {\bf r} ),\ {V_2}( {\bf r} ),\ {V_3}( {\bf r} ),\ {V_4}( {\bf r} )} \}$ (${V_i}({\bf r})$ uniformly distributed within $[ { - W/2,\ W/2} ]$) where disorder strength *W* comparable with ${\epsilon _{12}},\ {\epsilon _{34}}$ but much smaller than the topological gap ${{\rm{\Delta }}_b}$. On the contrary, the non-Abelian braiding remains integrity until the disorder is strong enough to destruct the topological gap [Fig. 3(d)], if the disorder has a chiral-symmetry-conserved form as $H_{\rm dis}^C = \ {\rm diag}\{ {{V_1}( {\bf r} ),\ {V_2}( {\bf r} ),\ - {V_2}( {\bf r} ),\ - {V_1}( {\bf r} )} \}$ (see Supplementary Material).

Due to the self-conjugation condition $\gamma _i^\dagger = {\gamma _i}\ $, MZM’s occupation energy term $\gamma _i^\dagger \ {\gamma _i} = \ 1$ is a trivial constant. For Jackiw-Rebbi zero-mode, however, $\psi _i^\dagger \ne {\psi _i}$ and the energy deviation ${{\rm{\Delta }}_{2i - 1,2i}}\psi _i^\dagger {\psi _i}$ (e.g., originated from disorder effect or gate voltages tuning local chemical potential) can be introduced into the Hamiltonian as:

(4) \begin{eqnarray*}{H_{\rm JR}} &=& {{\rm{\Delta }}_{12}}{\rm{\ }}\psi _1^\dagger {\psi _1} - {{\rm{\Delta }}_{12}}\psi _2^\dagger {\psi _2} + {{\rm{\Delta }}_{34}}\psi _3^\dagger {\psi _3}\nonumber\\ && -\, {{\rm{\Delta }}_{34}}\psi _4^\dagger {\psi _4} + \left( {\epsilon _{12}}{e^{i{\alpha _{12}}}}\psi _1^\dagger {\psi _2}\right.\nonumber\\ &&\left. +\, {\epsilon _{34}}{e^{i{\alpha _{34}}}}\psi _4^\dagger {\psi _3} + h.c. \right)\end{eqnarray*}

(widely separated zero-modes ${\psi _5}$ and ${\psi _6}$ with negligible coupling are dropped). The eigenstates of Equation (4) spanned by $| {{\psi _1}} \rangle $ and $| {{\psi _2}} \rangle $ are $\psi _ \pm ^{12} = \frac{1}{{\sqrt 2 C_{12}^ \pm }}\ \{ {\psi _1} + {e^{ - i{\alpha _{12}}}}[ \pm ( {{\rm{\tilde{\Delta }}}_{12}^2 + 1{)^{1/2}} - {{{\rm{\tilde{\Delta }}}}_{12}}} ]{\psi _2}\} $ (${{\rm{\tilde{\Delta }}}_{12}} \equiv {{\rm{\Delta }}_{12}}/{\epsilon _{12}}$, and $C_{12}^ \pm $ are normalization constants). In case of non-zero ${{\rm{\Delta }}_{12}}$, numerical simulation confirms that the exchange properties ${\psi _2} \to {\psi _3}$ and ${\psi _3} \to - {\psi _2}$ are still valid (see Supplementary Material). However, due to the new form of eigenstates $\psi _ \pm ^{12}$ with different weights of ${\psi _1}$ and ${\psi _2}$ (in other words, ${{\rm{\tilde{\Delta }}}_{12}}$ induces a ‘rotation’ of the eigenstates $\psi _ \pm ^{12}$), a novel non-Abelian braiding is obtained:

(5) \begin{eqnarray*} \left| {\phi \left( {t = 6T} \right)} \right\rangle = - {\rm{sin\ }}\delta \cdot \left| {\psi _ - ^{12}} \right\rangle + {\rm{cos\ }}\delta \cdot \left| {\psi _ + ^{12}} \right\rangle,\nonumber\\ \end{eqnarray*}

where $| {\phi ( {t\ = \ 0} )} \rangle \ = \ | {\psi _ - ^{12}} \rangle $, and $\delta \in ( { - \pi /2, \pi /2} )$ is defined as ${\rm{sin\ }}\delta \equiv {{\rm{\tilde{\Delta }}}_{12}}/{({\rm{\tilde{\Delta }}}_{12}^2 + 1)^{1/2}}$ and ${\rm{cos\ }}\delta \equiv 1/{(\tilde {\Delta}_{12}^2 + 1)^{1/2}}$. With the increase of $| \delta |$ describing the degeneracy lifting, the $| {\psi _ - ^{12}} \rangle $ component in $| {\phi ( {t\ = \ 6T} )} \rangle $ increases from 0 to 1, while the weight of $| {\psi _ + ^{12}} \rangle $ decreases from 1 to 0. In the adiabatic condition ${{\rm{\Delta }}_b} \gg 1/T \gg {\epsilon _{12}},\ {\epsilon _{34}}$ [31], numerical simulation results are *T*-independent and well fitted by Equation (5) [Fig. 3(e)]. According to the model described above, if moderate coupling strength ${\epsilon _{2i - 1,2i}}$ between Jackiw-Rebbi zero-modes is provided, $\delta $ will be relatively small for weak disorder and therefore the non-Abelian braiding properties identical to the MZMs can be retrieved. Furthermore, by exerting additional gate voltages modulating the local chemical potentials of ${\psi _1}$ and ${\psi _4}$, $\delta $ and then the non-Abelian braiding properties can be modulated in a controlled manner. For chiral-symmetry-conserved disorder $H_{\rm dis}^C$, in contrast, disorders in opposite signs are imposed on edge states with opposite chirality, hence the energy deviation ${{\rm{\Delta }}_{12}} = \ 0$ and the corresponding non-Abelian properties remain the same as the MZM.

Mathematically, in the basis of $\{ | {\psi _1},\ {\psi _2}; \psi _ - ^{12} \rangle ,\ | {{\psi _1},\ {\psi _2};\ \psi _ + ^{12}} \rangle \}$ (where $| {{\psi _1},\ {\psi _2};\ \psi _ \pm ^{12}} \rangle $ indicating ${\psi _1}$ and ${\psi _2}$ fusing into $\psi _ \pm ^{12}$), as shown in Table 1, such novel braiding can be expressed in the form of braiding operator *B* [46,47]. Besides, the fusion operator *F* and the exchange operator *R* can also be extracted from $B\ = {F^{ - 1}}\ {R^2}F$ (see Supplementary Material). The fusion rule of Jackiw-Rebbi zero-mode can be tuned by $\delta $ and is a generalization of MZM (Table 1). In the limit of $\delta \ = \ 0$, as expected, it retrieves MZM’s fusion rule up to a phase ${\theta _B}$. Such generalized and tunable fusion rule implies potential application in topological quantum computation.

**Table 1.** Braiding operator ${{B\ }} = {{{F}}^{ - 1}}{{\ }}{{{R}}^2}{{F}}$, fusion operator *F*, and the square of exchange operator *R* for both Jackiw-Rebbi zero-mode and MZM. ${{{\theta }}_{{B}}}$ is an overall phase factor.

Operator	Jackiw-Rebbi zero-mode	MZM
*B*	${e^{ - i{\theta _B}}}\left( {\begin{array}{@{}*{2}{c}@{}} { - {\rm{sin\ }}\delta }&{{\rm{cos\ }}\delta }\\ {{\rm{cos\ }}\delta }&{{\rm{sin\ }}\delta } \end{array}} \right)$	${e^{ - i\frac{\pi }{4}}}\left( {\begin{array}{@{}*{2}{c}@{}} 0&1\\ 1&0 \end{array}} \right)$
*F*	$\left( {\begin{array}{@{}*{2}{c}@{}} { - {\rm{sin\ }}( {\frac{\delta }{2} - \frac{\pi }{4}} )}&{{\rm{cos\ }}( {\frac{\delta }{2} - \frac{\pi }{4}} )}\\ {{\rm{cos\ }}( {\frac{\delta }{2} - \frac{\pi }{4}} )}&{{\rm{sin\ }}( {\frac{\delta }{2} - \frac{\pi }{4}} )} \end{array}} \right)$	$\frac{1}{{\sqrt 2 }}\left( {\begin{array}{@{}*{2}{c}@{}} 1&1\\ 1&{ - 1} \end{array}} \right)$
${R^2}$	${e^{ - i{\theta _B}}}\left( {\begin{array}{@{}*{2}{c}@{}} 1&0\\ 0&{ - 1} \end{array}} \right)$	${e^{ - i\frac{\pi }{4}}}\left( {\begin{array}{@{}*{2}{c}@{}} 1&0\\ 0&{ - 1} \end{array}} \right)$

Finally, if ‘fictitious’ PH-symmetry-breaking disorder is introduced into a $p \pm ip$-wave superconductor (D symmetry class [48,49]), then similar $\delta $-dependent braidings are also observed for MZM (see Supplementary Material). The only difference is that the important role preserving MZMs’ degeneracy is played by PH symmetry instead of chiral symmetry.

## DISCUSSIONS

The relation and similarity between Jackiw-Rebbi and MZM are uncovered by both AB effect and non-Abelian braiding properties. Though the double-frequency AB oscillation of the Jackiw-Rebbi zero-mode is irrelevant to the charge fractionalization, such effect relies on the resonant condition $\tilde{E} = \ 0$ in which Jackiw-Rebbi zero-mode's zero-energy nature is topologically protected, while such peculiarity can be easily removed for an ordinary zero-mode such as a localized state in a quantum dot. As for the non-Abelian properties, the symmetry-protected degeneracy for MZM is robust since PH symmetry is always presented provided that the superconductivity is not destroyed, therefore the fusion rule of MZM has a fixed form while the Jackiw-Rebbi one is tunable. Furthermore, for MZM-based braiding, considering the adiabatic condition ${\epsilon _{12}},\ {\epsilon _{34}} \ll 1/T \ll {{\rm{\Delta }}_b}$ [31] where the SC gap ${{\rm{\Delta }}_b}$ is in the order of 1 meV, it requires relative low braiding frequency $1/T$ and larger device scale to reduce the finite-size-induced coupling ${\epsilon _{12}},\ {\epsilon _{34}}$. These restrictions could be relaxed for Jackiw-Rebbi zero-modes since superconductivity is no longer required and the topological gap ${{\rm{\Delta }}_b}$ could be generally larger. These comparisons show the possibility of quantum computation device with higher integration level and higher braiding frequency.

## Supplementary Material

nwz189_Supplemental_File
