# Supplementary material for: Double-frequency Aharonov-Bohm effect and non-Abelian braiding properties of Jackiw-Rebbi zero-mode
Source: Natl Sci Rev. 2019 Nov 22;7(3):572–8. doi: 10.1093/nsr/nwz189 (PMC8288965; doi:10.1093/nsr/nwz189)
Supplement: nwz189_Supplemental_File [file nwz189_supplemental_file.pdf]

# Supplementary Materials for "Double-frequency Aharonov-Bohm effect and non-Abelian braiding properties of Jackiw-Rebbi zero-mode"

Yijia Wu,[1] Haiwen Liu,[2] Jie Liu,[3,*] Hua Jiang,[4] and X. C. Xie[1,5,6,†]

[1]*International Center for Quantum Materials, School of Physics, Peking University, Beijing 100871, China*
[2]*Center for Advanced Quantum Studies, Department of Physics, Beijing Normal University, Beijing 100875, China*
[3]*Department of Applied Physics, School of Science, Xi'an Jiaotong University, Xi'an 710049, China*
[4]*School of Physical Science and Technology, Soochow University, Suzhou 215006, China*
[5]*Beijing Academy of Quantum Information Sciences, Beijing 100193, China*
[6]*CAS Center for Excellence in Topological Quantum Computation, University of Chinese Academy of Sciences, Beijing 100190, China*

## DERIVATION OF THE $S$-MATRIX FOR JACKIW-REBBI ZERO-MODE'S AHARONOV-BOHM EFFECT

The Hamiltonian describing an AB ring with a Jackiw-Rebbi zero-mode embedded in one arm has the form of:

$$H_{\text{AB}} = -iv_f \sum_{i=1,2} \int_{-\infty}^{+\infty} dx \cdot \psi_i^{\dagger}(x)\, \partial_x \psi_i(x) + t_d \left[ e^{i\phi} \psi_1^{\dagger}(0)\, \psi_2(0) + h.c. \right] + t_0 \sum_{i=1,2} \left[ \varphi^{\dagger}(0)\, \psi_i(0) + h.c. \right] + \epsilon_0 \varphi^{\dagger}(0)\, \varphi(0) \tag{S1}$$

where $i = 1, 2$ is the lead index. The creation operator for the conducting mode (in the metal lead) and the Jackiw-Rebbi zero-mode are denoted as $\psi_i^{\dagger}(x)$ and $\varphi^{\dagger}(0)$, respectively. From left to right, the four terms in Eq. (S1) are the kinetic energy of the metal leads ($v_f$ the Fermi velocity), direct hopping term (with strength $t_d$) between two metal leads, hopping term (with strength $t_0$) between the Jackiw-Rebbi zero-mode and the metal leads, and the on-site energy (denoted by $\epsilon_0$) of the Jackiw-Rebbi zero-mode, respectively.

The Hamiltonian Eq. (S1) is derivated as following. Assuming both these two metal leads in the AB ring contain only one conducting mode per moving direction. Hence the Hamiltonian of the first lead can be written as [S1]:

$$H_{L1} = \sum_{\epsilon=L,R} \sum_{\sigma=\uparrow,\downarrow} -iv_f \int_{0}^{+\infty} dx \cdot \psi_{1\epsilon\sigma}^{\dagger}(x)\, \partial_x \psi_{1\epsilon\sigma}(x) \tag{S2}$$

where $v_f$ is the Fermi velocity, $\epsilon$ denotes the left-/right-moving mode, and $\sigma$ is the spin index. Assuming $\psi_{1L\sigma}(x) = \psi_{1R\sigma}(-x)$ for $x > 0$ and suppressing the right-moving index $\epsilon = R$, Eq. (S2) is simplified as [S1]:

$$H_{L1} = -iv_f \sum_{\sigma=\uparrow,\downarrow} \int_{-\infty}^{+\infty} dx \cdot \psi_{1\sigma}^{\dagger}(x)\, \partial_x \psi_{1\sigma}(x) \tag{S3}$$

The Hamiltonian for the second lead $H_{L2}$ can be dealed with in the same way. Besides, in the AB ring, the two hopping paths between the tip of these two leads have the form of:

$$H_T = t_d \sum_{\sigma=\uparrow,\downarrow} \left[ e^{i\phi} \psi_{1\sigma}^{\dagger}(0)\, \psi_{2\sigma}(0) + h.c. \right] + \frac{t_0}{\sqrt{2}} \sum_{i=1,2} \sum_{\sigma=\uparrow,\downarrow} \left[ \xi_\sigma \varphi^{\dagger}(0)\, \psi_{i\sigma}(0) + h.c. \right] \tag{S4}$$

Both hopping strength $t_d$ and $t_0$ are assumed to be real, $\xi_\sigma$ are complex numbers with $|\xi_\sigma| = 1$ and $\phi$ is the magnetic flux inclosed. Operating a unitary transformation $\begin{cases} \psi_i(x) = \frac{1}{\sqrt{2}} \left[ \xi_\uparrow \psi_{i\uparrow}(x) + \xi_\downarrow \psi_{i\downarrow}(x) \right] \\ \psi_i^{'}(x) = \frac{1}{\sqrt{2}} \left[ \xi_\uparrow \psi_{i\uparrow}(x) - \xi_\downarrow \psi_{i\downarrow}(x) \right] \end{cases}$ (where $i = 1, 2$) and dropping $\psi_i^{'}$ for not participating in the interference (only contributing a conductance constant), finally we get the full Hamiltonian whose form is exactly Eq. (S1) by combining $H_{L1}$, $H_{L2}$, $H_T$ and the on-site energy of the zero-mode.

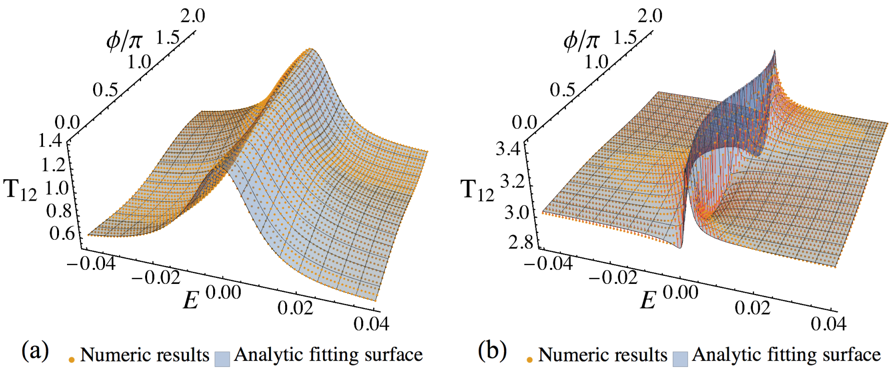


FIG. S1: (a) [(b)] The transmission coefficient $T_{12}$ as a function of the incident electron's energy $E$ and the magnetic flux $\phi$ inclosed, in the condition of weak (strong) $\tilde{t}_d$. Numerical results (obtained by the Green's function) are shown as orange dots, as the fitting formulae (by $S$-matrix) are shown as translucent blue surfaces. Red vertical lines indicate the fitting residues. The fitted parameters in (a) [(b)] are $\tilde{t}_d \approx 0.070$ ($\tilde{t}_d \approx 3.0$) and $\tilde{E} \approx 95E$ ($\tilde{E} \approx 122E$). Fig. 2 (b)-(d) [(e)-(g)] in the main text are replotted from the numerical results shown in (a) [(b)] of this figure.

Adopting the celebrated Heisenberg's equation of motion (EOM) $i\partial_t \hat{O} = [\hat{O}, H]$, we can write down the EOMs for $\psi_1$, $\psi_2$ and $\varphi$ with real space and time variables. Operating the Fourier transform (where $i = 1, 2$)

$$\begin{cases} \psi_{iE}(x) = \frac{1}{2\pi} \int dt \cdot \psi_i(x,t)\, e^{iEt} \\ \varphi_E(0) = \frac{1}{2\pi} \int dt \cdot \varphi(0,t)\, e^{iEt} \end{cases} \tag{S5}$$

and then integrating the EOMs around $x = 0$ $\{\varphi_E(0) = [\varphi_E(0^+) + \varphi_E(0^-)]/2$, and $\psi_{iE}(0) = [\psi_{iE}(0^+) + \psi_{iE}(0^-)]/2$ are inserted$\}$, finally two independent EOMs can be written in a matrix form as

$$\begin{pmatrix} 1 - i\tilde{t}_d e^{-i\phi} & -1 + i\tilde{t}_d e^{i\phi} \\ -\frac{i}{2} - \tilde{E} & -\frac{i}{2} - i\tilde{t}_d e^{i\phi}\tilde{E} \end{pmatrix} \begin{pmatrix} \psi_{1E}(0^+) \\ \psi_{2E}(0^+) \end{pmatrix} = \begin{pmatrix} 1 + i\tilde{t}_d e^{-i\phi} & -1 - i\tilde{t}_d e^{i\phi} \\ \frac{i}{2} - \tilde{E} & \frac{i}{2} + i\tilde{t}_d e^{i\phi}\tilde{E} \end{pmatrix} \begin{pmatrix} \psi_{1E}(0^-) \\ \psi_{2E}(0^-) \end{pmatrix} \tag{S6}$$

where $\tilde{t}_d \equiv \frac{t_d}{2v_f}$, and $\tilde{E} \equiv \frac{v_f}{t_0^2}(E - \epsilon_0)$. The operator at $x = 0^-$ ($x = 0^+$) is explained as the incoming (outgoing) mode, since the conducting mode of the lead at $x = 0^-$ is mapped from the left-moving mode. Therefore, the $S$-matrix defined as $\begin{pmatrix} \psi_{1E}(0^+) \\ \psi_{2E}(0^+) \end{pmatrix} = S \begin{pmatrix} \psi_{1E}(0^-) \\ \psi_{2E}(0^-) \end{pmatrix}$ has the explicit form of (where $\lambda \equiv i\tilde{t}_d e^{i\phi}$)

$$S = \frac{1}{\left(1 + \tilde{t}_d^2\right)\tilde{E} + \tilde{t}_d \cos\phi + i} \times \begin{pmatrix} \left(1 - \tilde{t}_d^2\right)\tilde{E} - \tilde{t}_d \cos\phi & -2\lambda \cdot \tilde{E} - i \\ 2\lambda^* \cdot \tilde{E} - i & \left(1 - \tilde{t}_d^2\right)\tilde{E} - \tilde{t}_d \cos\phi \end{pmatrix} \tag{S7}$$

and $T_{12}$ is the modulus square of the non-diagonal element of the $S$-matrix as $T_{12} = |S_{12}|^2$, which is exactly Eq. (2) in the main text. As shown in Fig. S1, the numerical results of $T_{12}$ (obtained by the Green's function) could be fitted by the analytic formula as $T_{12} = c_0 + c_1 \cdot \frac{4\tilde{t}_d^2 \cdot \tilde{E}^2 + 4\tilde{t}_d \cos(\phi+\phi') \cdot \tilde{E} + 1}{\left[\left(1+\tilde{t}_d^2\right)\tilde{E} + \tilde{t}_d \cos(\phi+\phi')\right]^2 + 1}$ [where $c_0$, $c_1$, and $\phi'$ are constants].

## DERIVATION OF THE $S$-MATRIX FOR MAJORANA ZERO-MODE'S AHARONOV-BOHM EFFECT

The Hamiltonian describing an AB ring with a Majorana zero-mode (MZM) embedded can be obtained by substituting the last two terms of Eq. (S1) by:

$$-it_M \cdot \eta(0) \sum_{i=1,2} [\psi_i(0) + h.c.] + \epsilon_M \cdot \eta^\dagger(0)\,\eta(0) \tag{S8}$$

where $\eta(0)$ is the MZM operator. The first term of Eq. (S8) is the coupling (with strength $t_M$) between MZM and the metal leads, as the second term is the on-site energy of the MZM.

The derivation of the $S$-matrix describing MZM's AB effect is in the same procedure as the Jackiw-Rebbi zero-mode's case. An important difference lies in that the EOMs for $\psi_i$ and $\psi_i^\dagger$ are coupled due to the presence of MZM. Therefore the $S$-matrix relates the incoming mode and outgoing mode has the definition of

$$\begin{pmatrix} \psi_{1E}(0^+) \\ \psi_{2E}(0^+) \\ \psi^\dagger_{1-E}(0^+) \\ \psi^\dagger_{2-E}(0^+) \end{pmatrix} = S \begin{pmatrix} \psi_{1E}(0^-) \\ \psi_{2E}(0^-) \\ \psi^\dagger_{1-E}(0^-) \\ \psi^\dagger_{2-E}(0^-) \end{pmatrix}, \; S \equiv \begin{pmatrix} S^{ee} & S^{eh} \\ S^{he} & S^{hh} \end{pmatrix} \tag{S9}$$

(where $1, 2$ for lead indices, and $e, h$ for electron and hole, respectively) in the Bogoliubov-de Gennes (BdG) basis. The explicit form of the $S$-matrix is shown to be:

$$S = C \begin{pmatrix} \frac{1-|\lambda|^4}{2}\tilde{E} + i(1+\lambda)(1-\lambda^*) & -i(1+\lambda)^2 - \lambda\left(1+|\lambda|^2\right)\cdot\tilde{E} & i\left(-1+\lambda^2\right) & -i(1-\lambda)(1-\lambda^*) \\ -i(1-\lambda^*)^2 + \lambda^*\left(1+|\lambda|^2\right)\cdot\tilde{E} & \frac{1-|\lambda|^4}{2}\tilde{E} + i(1+\lambda)(1-\lambda^*) & -i(1+\lambda)(1+\lambda^*) & i\left(-1+\lambda^{*2}\right) \\ i\left(-1+\lambda^{*2}\right) & -i(1-\lambda)(1-\lambda^*) & \frac{1-|\lambda|^4}{2}\tilde{E} + i(1-\lambda)(1+\lambda^*) & -i(1+\lambda^*)^2 - \lambda^*\left(1+|\lambda|^2\right)\cdot\tilde{E} \\ -i(1+\lambda)(1+\lambda^*) & i\left(-1+\lambda^2\right) & -i(1-\lambda)^2 + \lambda\left(1+|\lambda|^2\right)\cdot\tilde{E} & \frac{1-|\lambda|^4}{2}\tilde{E} + i(1-\lambda)(1+\lambda^*) \end{pmatrix} \tag{S10}$$

The prefactor $C = \frac{2}{(1+|\lambda|^2)\cdot\left[4i+(1+|\lambda|^2)\cdot\tilde{E}\right]}$, besides $\tilde{t}_d \equiv \frac{t_d}{2v_f}$, $\tilde{E} \equiv \frac{2v_f}{t_M^2}E$, and $\lambda \equiv i\tilde{t}_d e^{i\phi}$. With the MZM, the conductance $G_i$ is defined as the derivative of the current inside the $i$th lead [S2, S3] with respect to the bias $V$:

$$G_i = \frac{dI_i}{dV} = \frac{e^2}{h}\cdot\left\{1 - |S^{ee}_{i1}|^2 - |S^{ee}_{i2}|^2 + |S^{he}_{i1}|^2 + |S^{he}_{i2}|^2\right\} \tag{S11}$$

The conductance in Eq. (S11) can be decomposed into two parts as $G_i = G_{ii} - G_{ij}$ $(i \neq j)$, where $G_{ii} = \frac{e^2}{h}\{1 - |S^{ee}_{ii}|^2 + |S^{he}_{ii}|^2\}$ is induced by the current which flows out of lead $i$ and then flows back into lead $i$, and $G_{ij} = \frac{e^2}{h}\{|S^{ee}_{ij}|^2 - |S^{he}_{ij}|^2\}$ $(i \neq j)$ is proportional to the current which flows out of lead $j$ and then flows into lead $i$. Apart from the conductance between two leads $G_{ij}$ [Eq. (3) in the main text], the explicit form of the conductance $G_i$ is

$$G_1 = \frac{e^2}{h}\cdot\frac{16}{\left(1+\tilde{t}_d^2\right)^2\tilde{E}^2 + 16}\cdot\left[1 + \frac{2\tilde{t}_d}{\left(1+\tilde{t}_d^2\right)^2}\sin\phi\right] \tag{S12}$$

and

$$G_2 = \frac{e^2}{h}\cdot\frac{16}{\left(1+\tilde{t}_d^2\right)^2\tilde{E}^2 + 16}\cdot\left[1 - \frac{2\tilde{t}_d}{\left(1+\tilde{t}_d^2\right)^2}\sin\phi\right] \tag{S13}$$

It is easy to see that $G_1$ and $G_2$ are in an anticorrelated fashion [S3], and the total conductance $G = G_1 + G_2 = \frac{2e^2}{h}\cdot\frac{16}{\left(1+\tilde{t}_d^2\right)^2\cdot\tilde{E}^2+16}$ is quantized at $\frac{2e^2}{h}$ in the zero-energy condition [S3]. The total conductance $G$ decays in the manner of $\frac{1}{1+E^2}$ for non-zero energy, and the oscillation term $\frac{e^2}{h}\cdot\frac{32\tilde{t}_d\cdot\sin\phi}{\left(1+\tilde{t}_d^2\right)\cdot\left[\left(1+\tilde{t}_d^2\right)^2\cdot\tilde{E}^2+16\right]}$ is always in the period of $2\pi$.

## NUMERICAL SIMULATION FOR THE BRAIDING OF JACKIW-REBBI ZERO-MODES

The Hamiltonian describing QSHI constriction [Eq. (1)] in a square lattice has the form of

$$H_0 = \sum_i \psi^\dagger_{\mathbf{r}_i} T_0 \psi_{\mathbf{r}_i} + \psi^\dagger_{\mathbf{r}_i} T_x \psi_{\mathbf{r}_i+\delta\hat{\mathbf{x}}} + \psi^\dagger_{\mathbf{r}_i} T_y \psi_{\mathbf{r}_i+\delta\hat{\mathbf{y}}} + h.c. \tag{S14}$$

where $\mathbf{r}_i$ stands for the location of the $i$th lattice site. $T_0$, $T_x$, and $T_y$ are the on-site energy, hopping term along the $x$-direction, and hopping term along the $y$-direction, respectively. Each of the four arms in the cross-shaped junction

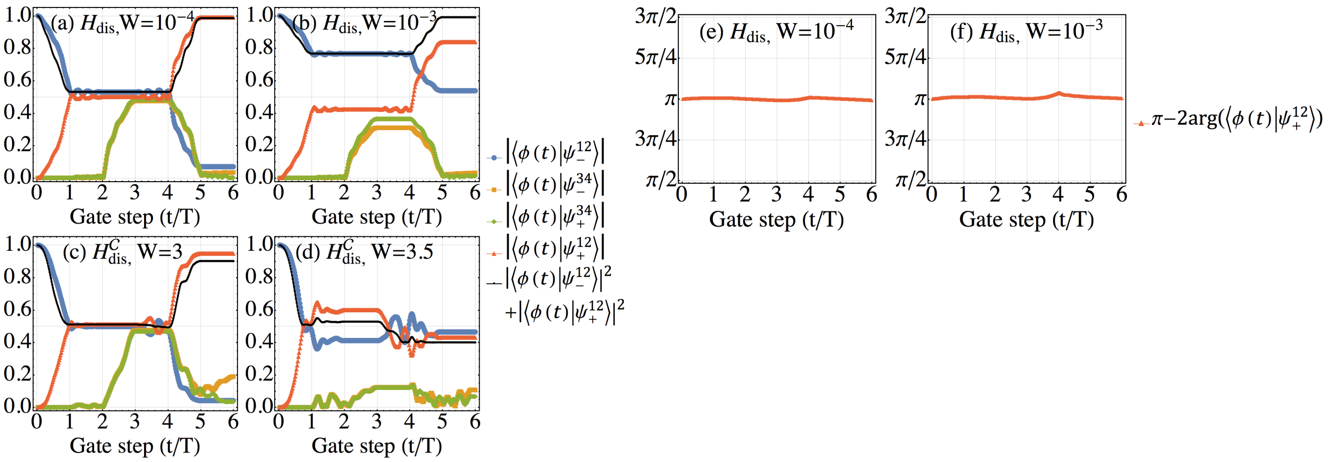


FIG. S2: Evolution of the eigenstate $|\phi(t)\rangle$ as two Jackiw-Rebbi zero-modes $\psi_2$ and $\psi_3$ are swapped twice in succession in the presence of disorder. Each gate step takes time of $T = 200$, topological gap $\Delta_b \approx 0.2$, and the coupling energy between Jackiw-Rebbi zero-modes $\epsilon_{12}, \epsilon_{34} \approx 7 \times 10^{-5}$. In the presence of chiral-symmetry-breaking disorder $H_{\rm dis}$, the modulus and phase angle [Eq. (S17)] of the projection of $|\phi(t)\rangle$ with disorder strength $W = 10^{-4}$ ($W = 10^{-3}$) are shown in (a) [(b)] and (e) [(f)], respectively. In the presence of chiral-symmetry-conserved disorder $H^C_{\rm dis}$, the modulus of the projection of $|\phi(t)\rangle$ with disorder strength $W = 3$ ($W = 3.5$) is shown in (c) [(d)]. The braiding property $|\psi^{12}_-\rangle \to |\psi^{12}_+\rangle$ nearly remains integrity in (a) and (c), while the novel non-Abelian braiding $|\psi^{12}_-\rangle \to -\sin\delta\cdot|\psi^-_{12}\rangle + \cos\delta\cdot|\psi^+_{12}\rangle$ is exhibited in (b). $|\langle\phi(t=6T)|\psi^{12}_-\rangle|^2 + |\langle\phi(t = 6T)|\psi^{12}_+\rangle|^2$ is significantly smaller than 1 in (d), as the non-Abelian braiding is destructed for strong disorder destroying the topological gap.

[Fig. 3 (a) in the main text] can be described by Eq. (S14), while the hopping term near the crossing controlled by the gate voltages has the form of

$$H_{\rm gate} = \sum_{\langle i,j\rangle} \left( \sum_{\alpha=1,2,3} g_\alpha \psi^\dagger_{\mathbf{r}_{i,\alpha}} T_x \psi_{\mathbf{r}_{j,c}} + \psi^\dagger_{\mathbf{r}_{i,4}} T_x \psi_{\mathbf{r}_{j,c}} + h.c. \right) \tag{S15}$$

where $\mathbf{r}_{i,\alpha}$ denotes the $i$th lattice site in the $\alpha$th arm ($\alpha = 1, 2, 3, 4$), $\mathbf{r}_{j,c}$ denotes the $j$th lattice site at the crossing point, and $\langle i, j\rangle$ means the nearest neighbour. In the numerical simulation, gate voltages G1, G2, G3 are turned on (off) linearly, therefore $g_\alpha$ ($\alpha = 1, 2, 3$) in Eq. (S15) is approximated as step functions $g_\alpha = 1 - n/N$ ($g_\alpha = n/N$) with $n = 0, 1, 2, ..., N$ ($N$ a large integer).

The whole braiding Hamiltonian $H_t = H_0 + H_{\rm gate}$ is time-dependent and the time-evolution operator in the form of $U(t) = \hat{\rm T}e^{i\int {\rm d}t\cdot H(t)}$ ($\hat{\rm T}$ is the time-ordering operator) is approximated as $U(t) \approx \prod_n e^{i\delta t\cdot H_t}$ due to the step-function approximation. The eigenstate of the junction evolves as $|\phi(t)\rangle = U(t)|\phi(t = 0)\rangle$ where $|\phi(t = 0)\rangle$ is the initial eigenstate (before braiding). As the braiding protocol stated in the main text, each braiding step takes time of $T$. The adiabatic condition is satisfied when the excitation energy $\sim 1/T$ will not give rise to energy level transition. There are two energy scales in the QSHI cross-shaped junction, the topological gap $\Delta_b$, and the coupling between Jackiw-Rebbi zero-modes $\epsilon_{12}, \epsilon_{34}$. In both Fig. 3 (b)-(d) in the main text and Fig. S2, $\Delta_b \approx 0.2$ and $\epsilon_{12}, \epsilon_{34} \approx 7\times10^{-5}$, so we choose $\delta_t = 0.1$ and $N = 1000$, hence the time cost in each braiding step $T = 2 \times N\delta_t = 200$ satisfies the adiabatic condition as $\Delta_b \gg 1/T \gg \epsilon_{12}$ [S6].

In the presence of chiral-symmetry-breaking disorder $H_{\rm dis} = {\rm diag}\{V_1(\mathbf{r}), V_2(\mathbf{r}), V_3(\mathbf{r}), V_4(\mathbf{r})\}$, as shown in Fig. S2 (a), (b), though the braiding results are significantly altered for disorder strength $W$ comparable with $\epsilon_{12}, \epsilon_{34}$ but much smaller than the topological gap $\Delta_b$, the equality $|\langle\phi(t = 6T)|\psi^{12}_-\rangle|^2 + |\langle\phi(t = 6T)|\psi^{12}_+\rangle|^2 = 1$ is still satisfied [where the eigenstate before braiding $|\phi(t = 0)\rangle = |\psi^{12}_-\rangle$]. Therefore, the eigenstate after braiding $|\phi(t = 6T)\rangle$ still lives in the Hilbert space spanned by $|\psi_1\rangle$ and $|\psi_2\rangle$ (other than mix with other states such as $|\psi_3\rangle$), in other words, $|\phi(t = 6T)\rangle$ is a superposition of $|\psi^{12}_-\rangle$ and $|\psi^{12}_+\rangle$. It is quite reasonable to assume that in the presence of disorder, the whole braiding process swapping $\psi_2$ and $\psi_3$ twice in succession give rise to $\psi_2 \to e^{-i\theta}\psi_2$, i.e., $|\phi(t = 6T)\rangle = \frac{1}{\sqrt{2}C^-_{12}}\{|\psi_1\rangle + e^{i\alpha_{12}}[-(\widetilde{\Delta}^2_{12} + 1)^{1/2} - \widetilde{\Delta}_{12}]e^{i\theta}\cdot|\psi_2\rangle\}$. As a result,

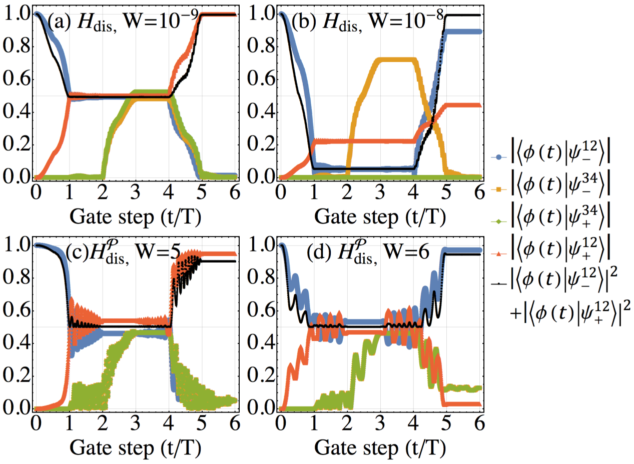


FIG. S3: Evolution of the eigenstate $|\phi(t)\rangle$ as two MZMs $\gamma_2$ and $\gamma_3$ are swapped twice in succession in the presence of disorder. Each gate step takes time of $T = 100$, SC gap $\Delta_{\rm SC} \approx 2.6$, and the coupling energy between MZMs $\epsilon_{12}, \epsilon_{34} \approx 1 \times 10^{-9}$. (a), (b) PH-symmetry-breaking disorder $H_{\rm dis}$ with disorder strength (a) $W = 10^{-9}$, and (b) $W = 10^{-8}$; (c), (d) PH-symmetry-conserved disorder $H^{\mathcal{P}}_{\rm dis}$ with disorder strength (c) $W = 5$, and (d) $W = 6$.

$$\langle\phi(t = 6T)|\psi^{12}_{+}\rangle = \frac{1 - e^{-i\theta}}{2\sqrt{\widetilde{\Delta}^2_{12} + 1}} = \frac{\sin{(\theta/2)}}{\sqrt{\widetilde{\Delta}^2_{12} + 1}} \cdot e^{i\frac{\pi-\theta}{2}} \tag{S16}$$

and therefore

$$\theta = \pi - 2\arg\langle\phi(t = 6T)|\psi^{12}_{+}\rangle \tag{S17}$$

$\theta = \pi$ is verified by the numerical results [Fig. S2 (e), (f)]. Consequently, the braiding property $\psi_2 \to -\psi_2$ after swapping $\psi_2$ and $\psi_3$ twice in succession is still valid even in the presence of weak disorder. Similarly, the braiding property $\psi_3 \to -\psi_3$ can be demonstrated in the same way. These properties indicate that swapping $\psi_2$ and $\psi_3$ once lead to the general form of $\psi_2 \to e^{i\theta_1}\psi_3$ and $\psi_3 \to e^{i\theta_2}\psi_2$ where $e^{i\theta_1}e^{i\theta_2} = -1$. Adopting a gauge transformation imposing $e^{i\theta_1} = 1$, then the braiding properties $\psi_2 \to \psi_3$ and $\psi_3 \to -\psi_2$ are identical to the MZM [S5].

On the contrary, as shown in Fig. S2 (c), (d), if the on-site disorder has the chiral-symmetry-conserved form as $H^{\mathcal{C}}_{\rm dis} = {\rm diag}\{V_1(\mathbf{r}), V_2(\mathbf{r}), -V_2(\mathbf{r}), -V_1(\mathbf{r})\}$ (satisfying $-H^{\mathcal{C}}_{\rm dis} = \mathcal{C}H^{\mathcal{C}}_{\rm dis}\mathcal{C}^{-1}$), then the non-Abelian properties are preserved until the disorder is strong enough to close the topological gap. $|\langle\phi(t = 6T)|\psi^{12}_{-}\rangle|^2 + |\langle\phi(t = 6T)|\psi^{12}_{+}\rangle|^2$ is significantly smaller than 1 in Fig. S2 (d), indicating the gap is destructed and the Jackiw-Rebbi zero-modes are mixed with the bulk states.

## BRAIDING PROPERTIES OF MAJORANA ZERO-MODES IN THE PRESENCE OF DISORDER

MZMs' braiding is performed with the same protocol and in the same shape of junction as Jackiw-Rebbi zero-modes, where the difference is that the cross-shaped junction here is composed of $p \pm ip$-wave SC supporting MZMs. The Hamiltonian of $p \pm ip$-wave SC in the BdG basis

$$H_{p\pm ip{\rm SC}}(\mathbf{p}) = \frac{1}{2} \times (c^\dagger_{\mathbf{p}}, c_{-\mathbf{p}}) \left[\Delta(p_x\sigma_x + p_y\sigma_y) + (\frac{\mathbf{p}^2}{2m} - \mu)\sigma_z\right] \begin{pmatrix} c_{\mathbf{p}} \\ c^\dagger_{-\mathbf{p}} \end{pmatrix} \tag{S18}$$

possesses PH symmetry as $-H(-\mathbf{p}) = \mathcal{P}H^T(\mathbf{p})\mathcal{P}^{-1}$ with $\mathcal{P} = \sigma_x$, and hence is in the D symmetry class (the same symmetry class as Kitaev's chain with complex SC pairing).

Similar to the Jackiw-Rebbi case, there are six MZMs (denoted as $\gamma_{i=1,2,...,6}$) in the cross-shaped junction, and the effective Hamiltonian describing the coupling energy ($\epsilon_{2i-1,2i}$) and a "fictitious" energy deviation ($\Delta_{2i-1,2i}$) of the MZMs reads [S4]:

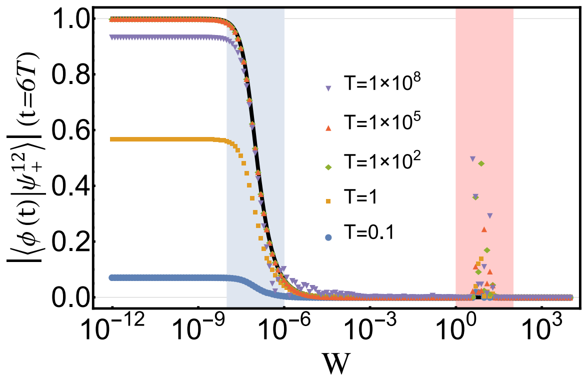


FIG. S4: Numerical simulation results of $|\langle\phi(t=6T)|\psi_+^{12}\rangle|$ in a fixed PH-symmetry-breaking disorder profile with different disorder strength $W$ and braiding time $T$. The coupling energy between MZMs $\epsilon_{12}, \epsilon_{34} \approx 1\times 10^{-9}$, and the SC gap $\Delta_{\rm SC} \approx 2.6$. Too long ($T = 1\times 10^8$) or too short ($T = 0.1, 1$) braiding time will violate the adiabatic condition $\Delta_{\rm SC} \gg 1/T \gg \epsilon_{12}, \epsilon_{34}$. The fitting curve for the adiabatic results ($T = 1\times 10^2, 1\times 10^5$) by Eq. (S20) is shown in black. The blue (red) shaded region indicates $W \sim \epsilon_{12}, \epsilon_{34}$ ($W \sim \Delta_{\rm SC}$).

$$H_{\rm M} = i\epsilon_{12}\gamma_1\gamma_2 + i\epsilon_{34}\gamma_4\gamma_3 + \Delta_{12}\gamma_1\gamma_1 - \Delta_{12}\gamma_2\gamma_2 + \Delta_{34}\gamma_3\gamma_3 - \Delta_{34}\gamma_4\gamma_4 \tag{S19}$$

where the two widely separated MZMs $\gamma_5$ and $\gamma_6$ are neglected. Two eigenstates of Eq. (S19) formed by $\gamma_1$ and $\gamma_2$ are $\psi_\pm^{12} = \frac{1}{\sqrt{2C_{12}^\pm}}\{\gamma_1 + i[\widetilde{\Delta}_{12} \mp (1+\widetilde{\Delta}_{12}^2)^{1/2}]\gamma_2\}$ (where $\widetilde{\Delta}_{12} \equiv \Delta_{12}/\epsilon_{12}$, and $C_{12}^\pm$ are normalization constants). Swapping two MZMs $\gamma_2$ and $\gamma_3$ lead to $\gamma_2 \to \gamma_3$ and $\gamma_3 \to -\gamma_2$ [S5], therefore a full braiding process swapping $\gamma_2$ and $\gamma_3$ twice in succession gives rise to $\gamma_2 \to -\gamma_2$ and $\gamma_3 \to -\gamma_3$. After the whole braiding process, it can be shown that

$$|\phi(t=6T)\rangle = -\sin\delta\cdot|\psi_-^{12}\rangle + \cos\delta\cdot|\psi_+^{12}\rangle \tag{S20}$$

[where $|\phi(t=0)\rangle = |\psi_-^{12}\rangle$, $\sin\delta \equiv \widetilde{\Delta}_{12}/(\widetilde{\Delta}_{12}^2+1)^{1/2}$ and $\cos\delta \equiv 1/(\widetilde{\Delta}_{12}^2+1)^{1/2}$], whose form is exactly the same as the Jackiw-Rebbi zero-modes with lifted degeneracy [Eq. (5) in the main text]. It is worth noting that if the MZM's degeneracy is presented ($\widetilde{\Delta}_{12} = 0$), then $\psi_-^{12} = \frac{1}{\sqrt{2}}(\gamma_1 + i\gamma_2) = (\psi_+^{12})^\dagger$ and a basis of fermion occupation number $\Big(|0\rangle, \Psi_1^\dagger|0\rangle, \Psi_2^\dagger|0\rangle, \Psi_1^\dagger\Psi_2^\dagger|0\rangle\Big)$ is usually adopted [S4–S6] instead of $\big(|\psi_-^{12}\rangle, |\psi_-^{34}\rangle, |\psi_+^{34}\rangle, |\psi_+^{12}\rangle\big)$.

The braiding of MZMs in the $p\pm ip$-wave SC and the corresponding evolution of the eigenstates can be numerically simulated in the same way as the Jackiw-Rebbi case. Though it might be experimentally unrealistic, a "fictitious" PH-symmetry-breaking disorder in the form of $H_{\rm dis} = {\rm diag}\{V_1(\mathbf{r}), V_2(\mathbf{r})\}$ with $V_i(\mathbf{r}) \in [-W/2, W/2]$ still can be introduced in the numerical simulation, and similar novel non-Abelian braiding is observed at weak disorder $W \ll \Delta_{\rm SC}$ [Fig. S3 (a), (b), where $\Delta_{\rm SC}$ is the SC gap]. Moreover, as shown in Fig. S4, we numerically investigate $|\langle\phi(t=6T)|\psi_+^{12}\rangle|$ in a fixed disorder profile with different disorder strength $W$ and braiding time $T$. Too long (too short) braiding time $T$ will induce energy level transition between different MZMs (between MZMs and bulk states) as the energy level transition $(\epsilon_{\rm f} - \epsilon_{\rm i}) \sim 1/T$. For intermediate $T$ satisfying the adiabatic condition $\Delta_{\rm SC} \gg 1/T \gg \epsilon_{12}, \epsilon_{34}$, the braiding results are $T$-independent and can be perfectly fitted by Eq. (S20) as $|\langle\phi(t=6T)|\psi_+^{12}\rangle| = \big(1+aW^2\big)^{-1/2}$ (black curve in Fig. S4, $a$ is a fitting constant) since $\widetilde{\Delta}_{12} \propto W$ in the fixed disorder profile.

In analogy with the case of Jackiw-Rebbi zero-mode, in the condition that the disorder has a PH-symmetry-conserved form as $H_{\rm dis}^{\mathcal{P}} = V_1(\mathbf{r})\sigma_z$ [satisfying $-H_{\rm dis}^{\mathcal{P}} = \mathcal{P}(H_{\rm dis}^{\mathcal{P}})^T\mathcal{P}^{-1}$], MZMs' non-Abelian properties remain unchanged until the SC gap is destructed by strong disorder $W \sim \Delta_{\rm SC}$ [Fig. S3 (c), (d)]. This is quite reasonable since $H_{\rm dis}^{\mathcal{P}}$ imposes disorder of opposite signs on the electron band and hole band of the $p\pm ip$-wave SC, therefore the energy deviation of MZMs ($\Delta_{2i-1,2i}$) vanishes and the non-Abelian braiding properties maintain integrity.

## DERIVATION OF JACKIW-REBBI ZERO-MODE'S FUSION RULE

For the novel non-Abelian braiding of Jackiw-Rebbi zero-modes, as stated in Eq. (5) in the main text, an eigenstate $\phi(t)$ satisfying $|\phi(t=0)\rangle = |\psi_-^{12}\rangle$ will evolve into $|\phi(t=6T)\rangle = -\sin\delta\cdot|\psi_{12}^-\rangle + \cos\delta\cdot|\psi_{12}^+\rangle$, where $\delta \in (-\pi/2, \pi/2)$ is

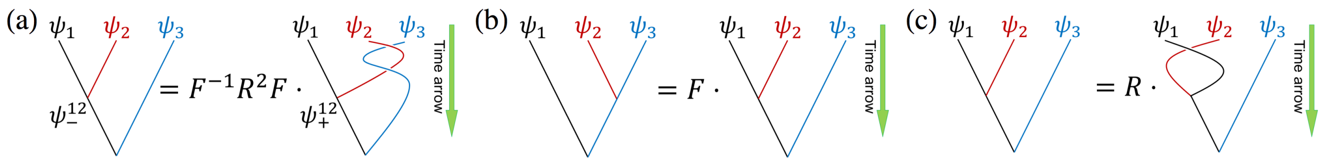


FIG. S5: Diagrammatic expression of the (a) braiding operation $B$, (b) fusion operation $F$, and (c) exchange operation $R$. The braiding operation $B$ can be decomposed as $B = F^{-1}R^2F$.

defined as $\sin\delta \equiv \widetilde{\Delta}_{12}/(\widetilde{\Delta}_{12}^2+1)^{1/2}$ and $\cos\delta \equiv 1/(\widetilde{\Delta}_{12}^2+1)^{1/2}$. Similarly, for another eigenstate $\varphi(t)$ initially in the state of $|\varphi(t=0)\rangle = |\psi_+^{12}\rangle$, we have $|\varphi(t=6T)\rangle = \cos\delta\cdot|\psi_{12}^-\rangle + \sin\delta\cdot|\psi_{12}^+\rangle$ after braiding.

The whole braiding process that swapping $\psi_2$ and $\psi_3$ twice in succession can be diagrammatically expressed as Fig. S5(a) [S7, S8]. The braiding operation $B$ can be decomposed by the fusion operator $F$ relating different fusion orders [Fig. S5(b)] and exchange operator $R$ describing the exchange of two Jackiw-Rebbi zero-modes [Fig. S5(c)] as $B = F^{-1}R^2F$. Therefore, in the basis of $\left\{|\psi_1,\psi_2;\psi_-^{12}\rangle,\ |\psi_1,\psi_2;\psi_+^{12}\rangle\right\}$, the braiding operator $B$ has the explicit form of:

$$B = e^{-i\theta_B}\begin{pmatrix} -\sin\delta & \cos\delta \\ \cos\delta & \sin\delta \end{pmatrix} \tag{S21}$$

where $\theta_B$ is an overall phase factor. The fusion operator $F$ is a unitary matrix that generally has the form as $F = \begin{pmatrix} w & z \\ -e^{i\lambda}z^* & e^{i\lambda}w^* \end{pmatrix}$ ($w, z$ are complex numbers satisfying $|w|^2+|z|^2=1$). We also reasonably assume that $R$ matrix has a diagonal form as $R = e^{-i\theta_R/2}\begin{pmatrix} 1 & 0 \\ 0 & e^{i\varphi/2} \end{pmatrix}$, indicating the exchange of two Jackiw-Rebbi zero-modes $\psi_1$ and $\psi_2$ generally induces different phases for $\psi_-^{12}$ and $\psi_+^{12}$. Therefore, the identical relation $FB = R^2F$ gives rise to:

$$\begin{cases} z\cos\delta = w\left[e^{-i(\theta_R-\theta_B)} + \sin\delta\right] \\ w\cos\delta = z\left[e^{-i(\theta_R-\theta_B)} - \sin\delta\right] \\ w\cos\delta = z\left[-e^{i(\theta_R-\theta_B)}e^{-i\varphi} - \sin\delta\right] \\ z\cos\delta = w\left[-e^{i(\theta_R-\theta_B)}e^{-i\varphi} + \sin\delta\right] \end{cases} \tag{S22}$$

It can be easily seen from Eq. (S22) that $e^{-i\cdot 2(\theta_R-\theta_B)} = \cos^2\delta+\sin^2\delta = 1$ and $e^{-i\varphi} = -e^{-i\cdot 2(\theta_R-\theta_B)} = -1$. Hence the $R$ matrix still has the form of $R^2 = e^{-i\theta_R}\begin{pmatrix} 1 & 0 \\ 0 & -1 \end{pmatrix}$, which is $\delta$-independent and identical to the MZM up to an overall phase factor $\theta_R$. Besides, there are two solutions corresponding to different roots of $e^{-i\cdot 2(\theta_R-\theta_B)} = 1$:

SOLUTION I: $e^{-i(\theta_R-\theta_B)} = -1$, $F = \begin{pmatrix} \cos\left(\frac{\delta}{2}-\frac{\pi}{4}\right)e^{-i\theta_w} & \sin\left(\frac{\delta}{2}-\frac{\pi}{4}\right)e^{-i\theta_w} \\ -e^{i\lambda}\sin\left(\frac{\delta}{2}-\frac{\pi}{4}\right)e^{i\theta_w} & e^{i\lambda}\cos\left(\frac{\delta}{2}-\frac{\pi}{4}\right)e^{i\theta_w} \end{pmatrix}$.

SOLUTION II: $e^{-i(\theta_R-\theta_B)} = 1$, $F = \begin{pmatrix} -\sin\left(\frac{\delta}{2}-\frac{\pi}{4}\right)e^{-i\theta_w} & \cos\left(\frac{\delta}{2}-\frac{\pi}{4}\right)e^{-i\theta_w} \\ -e^{i\lambda}\cos\left(\frac{\delta}{2}-\frac{\pi}{4}\right)e^{i\theta_w} & -e^{i\lambda}\sin\left(\frac{\delta}{2}-\frac{\pi}{4}\right)e^{i\theta_w} \end{pmatrix}$.

where $\theta_w = -\arg w$ is inserted. Noticing that these two solutions can actually be related by another unitary matrix $U = \begin{pmatrix} 0 & 1 \\ -1 & 0 \end{pmatrix}$. Finally, if we choose $e^{-i(\theta_R-\theta_B)} = 1$, $\theta_w = 0$ and $e^{i\lambda} = -1$ (the same as the MZM), then the fusion operator $F$ has a simplified form as:

$$F = \begin{pmatrix} -\sin\left(\frac{\delta}{2}-\frac{\pi}{4}\right) & \cos\left(\frac{\delta}{2}-\frac{\pi}{4}\right) \\ \cos\left(\frac{\delta}{2}-\frac{\pi}{4}\right) & \sin\left(\frac{\delta}{2}-\frac{\pi}{4}\right) \end{pmatrix} \tag{S23}$$

In the clean limit that $\delta = 0$, it retrieves the familiar expression of $F = \frac{1}{\sqrt{2}}\begin{pmatrix} 1 & 1 \\ 1 & -1 \end{pmatrix}$ identical to the case of MZM.

* Corresponding author: jieliuphy@xjtu.edu.cn
† Corresponding author: xcxie@pku.edu.cn

[S1] K. T. Law, P. A. Lee, and T. K. Ng, Phys. Rev. Lett. **103**, 237001 (2009).
[S2] J. Nilsson, A. R. Akhmerov, and C. W. J. Beenakker, Phys. Rev. Lett. **101**, 120403 (2008).
[S3] K. M. Tripathi, S. Das, and S. Rao, Phys. Rev. Lett. **116**, 166401 (2016).
[S4] C.-Z. Chen, Y.-M. Xie, J. Liu, P. A. Lee, and K. T. Law, Phys. Rev. B **97**, 104504 (2018).
[S5] D. A. Ivanov, Phys. Rev. Lett. **86**, 268 (2001)
[S6] C. S. Amorim, K. Ebihara, A. Yamakage, Y. Tanaka, and M. Sato, Phys. Rev. B **91**, 174305 (2015).
[S7] V. Lahtinen and J. Pachos, SciPost Physics **3**, 021 (2017).
[S8] J. K. Pachos, *Introduction to topological quantum computation* (Cambridge University Press, 2012)
